# Readmissions Following Pancreaticoduodenectomy: Experience From a Tertiary Care Center in India

**DOI:** 10.7759/cureus.65140

**Published:** 2024-07-22

**Authors:** Rajneesh Kumar Singh, Krishna Rao Gurana

**Affiliations:** 1 Department of Surgical Gastroenterology, Sanjay Gandhi Postgraduate Institute of Medical Sciences (SGPGIMS), Lucknow, IND

**Keywords:** pancreaticoduodenectomy (pd), sequela of surgery, intervention, outcome, readmissions

## Abstract

Background

An enhanced recovery approach in surgery helps early postoperative discharge. With the decreasing trend of morbidity and mortality in recent times in patients undergoing complex procedures such as pancreaticoduodenectomy, readmissions are the next major concern. The causes and outcomes of these readmissions should be investigated for their impact on patient care and prevention.

Methodology

A total of 997 patients discharged after pancreaticoduodenectomy from a tertiary care center in northern India, between 1989 and 2021, were studied retrospectively to assess the readmission rate for sequelae after pancreaticoduodenectomy. The causes, interventions, outcomes, and predictive factors were studied.

Results

A total of 103 (10.3%) patients required readmission for sequelae after pancreaticoduodenectomy, and 52 (50.4%) patients required interventions. The most common cause for readmission in our study was intra-abdominal collection (n = 23, 22.3%). Of these 103 patients, 63 (61.2%) had good outcomes, 36 (34.9%) had fair outcomes, and four (3.9%) had bad outcomes. Overall, 53 (51.5%) of 103 patients were readmitted within 30 days of discharge, most commonly with intra-abdominal collection (16 of 53, 30.1%). Of these 53 patients, 22 (41.5%) required interventions, 34 (64.1%) had good outcomes, and 27 (50.9%) were readmitted within seven days of discharge. Of these 27 patients, 12 (44.4%) required interventions, with 24 (88.8%) experiencing good outcomes. Of the 103 patients, 12 (11.6%) were readmitted between 31 and 90 days, mostly due to external stent, T-tube, or percutaneous transhepatic biliary drainage-related problems. Overall, 38 (36.9%) of 103 patients were readmitted after 90 days, mostly with incisional hernia and strictured hepaticojejunostomy. Of these 38 patients, 26 (68.4%) required intervention, and 23 (60.5%) had good outcomes. A previous history of cholangitis (odds ratio (OR) = 1.771, 95% confidence interval (CI) = 1.17-2.67, p = 0.007), postoperative fever (OR = 1.628, 95% CI = 1.081-2.452, p = 0.02), wound infection (OR = 2.011, 95% CI = 1.332-3.035, p = 0.001), and wound dehiscence (OR = 2.136, 95% CI = 1.333-3.423, p = 0.002) predicted readmission on univariate analysis. Multivariate analysis showed a previous history of cholangitis (OR = 1.755, CI = 1.158-2.659, p = 0.008) and wound infection (OR = 1.995, 95% CI = 1.320-2.690, p = 0.001) as factors independently predicting readmission.

Conclusions

Readmitted patients have high intervention rates and good recovery rates. Readmissions should not be considered a scale for poor healthcare. Patient education, proper management of postoperative complications, and a properly designed discharge care system can help tackle this problem.

## Introduction

Improved perioperative care has decreased the mortality and morbidity of surgical procedures. Mortality rates have decreased from 20% to 5% over the last few decades, and some institutes have documented near-zero mortality, defined as less than 2% [1,2]. Morbidity decreased from 40% to 23% in many centers [2]. Regionalization has contributed significantly to improving patient care for complex procedures such as pancreaticoduodenectomy (PD) [3,4]. Better understanding of the disease, enhanced imaging services, proper patient selection, advanced surgical equipment, enhanced perioperative care, and better management of postoperative complications have improved patients’ quality of life. Enhanced recovery approaches have aided in early postoperative discharge. In this context, readmissions become important because they decrease the quality of life, add anxiety to patients, and contribute to extra burden to the healthcare system [5]. A thorough understanding of the clinical profile of patients who are readmitted can throw light on the prevention of readmissions. In procedures such as PD, which has inbuilt high rates of morbidity, readmissions have a major impact. The primary aim and objective of this study is to analyze the incidence, causes, and outcomes of patients readmitted after PD because of surgical sequelae. In this study, we hypothesized that patient demographics, preoperative status, intraoperative details, and postoperative complications might have an association with readmissions following PD. The study also aimed at identifying factors that can predict readmissions and methods to address the problem.

The preliminary results of this paper were presented as a poster at HBP Surgery Week 2021 and the 54th Annual Congress of the Korean Association of HBP Surgery, virtually held on March 25-27, 2021, at the Grand Walkerhill Hotel, Seoul, Korea.

## Materials and methods

The study was approved by the Institutional Ethics Committee, Sanjay Gandhi Postgraduate Institute of Medical Sciences, Lucknow (2022-08-MCh-EXP-45 PGI/BE/152/2022). A total of 997 patients were discharged after PD (out of 1,085 patients who underwent PD) between 1989 and 2021 from a tertiary care center in northern India. Of the 997 patients, 898 (90.1%) had a malignant disease and 99 (9.9%) had a benign disease. Overall, 581 (58.3%) patients had major morbidity (Clavien-Dindo grade 2 or above); 159 (15.9%) patients suffered postoperative bleeding (both intraluminal and extraluminal); 640 (64.2%) had a postoperative pancreatic fistula (POPF) as per the International Study Group for Pancreatic Surgery; 327 (32.8%) had wound infection; 245 (24.6%) had delayed gastric emptying (DGE); and 73 (7.3%) had a hepaticojejunostomy (HJ) leak. The average postoperative stay was 18 days (SD = 12 days). A prospectively maintained hospital database was used to analyze different variables during the index hospitalization, readmission, and subsequent follow-up.

All patients who were discharged after PD (index surgery) from our institute and who required readmission for sequelae of surgery were included in the study retrospectively. Patients readmitted for tumor recurrence, chemotherapy-related causes, and readmissions unrelated to the index surgery were excluded. Patients readmitted to outside hospitals were also excluded.

Preoperative biliary drainage was indicated in patients with cholangitis, poor performance status, locally advanced disease, and high bilirubin as per the choice of the treating surgeon. Preoperative preparation of the patient, surgical technique, and postoperative management of complications were guided by departmental guidelines. These guidelines were regularly updated based on the recent literature available at that time. The definition and grading of complications was based on the updated International Study Group in Pancreatic Surgery guidelines at that time. Surgically placed intra-abdominal drains were analyzed according to the clinical status of the patients in the postoperative period. Intra-abdominal drain removal was done when the drain output was less than 30 mL per day and as per the decision of the treating surgeon based on the clinical condition of the patients. Patients were discharged postoperatively at the decision of the treating surgeon when the patients were afebrile (less than 99.5°F for two days), had stable inflammatory parameters on blood investigations, well-established nutrition, adequate pain control with oral analgesia, and compliant with the discharge advice at home.

Patients readmitted for sequelae of the index surgery were grouped based on the period of readmission: within 30 days of discharge, 31 to 90 days of discharge, and more than 90 days following discharge. The causes, interventions, and outcomes of each group were analyzed. Outcomes of readmissions were grouped as good (if both symptomatic and biochemical improvement were present), fair (if either symptomatic or biochemical improvement were present), and bad (if the condition of the patient deteriorated either symptomatically or biochemically).

Patients readmitted because of sequelae (group 1) of the index surgery were analyzed and compared with patients who were not readmitted (group 2) with respect to demographic features, clinical presentation, intraoperative details of the index surgery, disease pathology, and postoperative complications. Patients on regular medications for any of the diseases, i.e., coronary heart disease, valvular heart disease, pre-existing liver disease or renal disease, or lung parenchymal disease, were considered to have medical risk factors affecting mortality. Factors determining readmission were analyzed using univariate and multivariate analysis. Our institute’s experience with readmissions following PD for the study duration (1989-2021) was presented in a quinquennially time frame. Yearly readmission rates were also analyzed and presented appropriately.

Statistical analysis

Statistical analysis was done using SPSS Statistics Version 20 (IBM Corp., Armonk, NY, USA). Continuous variables were presented in mean ± SD, and a comparison of means between the two groups was done by an independent t-test. Categorical variables were presented as numbers (%). Univariate binary logistic regression analysis was used to identify the variables that were significantly associated with the readmission of the patients. All significant variables in univariate analysis were tested using the chi-square test for the multicollinearity and were not significantly associated with each other. These variables were further included in the multivariate binary logistic regression analysis. Unadjusted odds ratio (OR) and adjusted odds ratio along with a 95% confidence interval (CI) were used to present the results of the univariate and multivariate analysis respectively. P-values less than 0.05 were considered statistically significant.

## Results

Of the 997 patients, 103 (10.3%) were readmitted (group 1) because of sequelae related to the index surgery. These patients (group 1, n = 103) had an average postoperative hospital stay of 20.74 days (SD = 14.35) during their index surgery. Patients who were not readmitted (group 2, n = 894) had an average postoperative hospital stay of 17.74 days (SD = 11.61) with a p-value of 0.014. Table 1 enumerates the causes of readmissions due to sequelae of index surgery and the intervention requirement for each cause of readmission. The most common cause for readmission in our study was intra-abdominal collection (n = 23, 22.3%). Overall, 52 (50.4%) of 103 patients required intervention during readmission. Of these 103 readmitted patients, 63 (61.2%) had good outcomes, 36 (34.9%) had fair outcomes, and four (3.9%) had bad outcomes. The average hospital stay during readmission was 13 days (range = 1-150 days). No mortality was documented.

**Table 1:** Causes of readmissions due to sequelae of index surgery and intervention requirement in each cause of readmission. SAIO = subacute intestinal obstruction; PTBD = percutaneous transhepatic biliary drainage; POPH = postoperative pancreatic hemorrhage; DGE = delayed gastric emptying; HJ = hepaticojejunostomy

Causes of readmissions	Frequency (n = 103)	Intervention requirement in each cause of readmission (n = frequency in that particular cause, by %)
Intra-abdominal collection	23 (22.3%)	13 (56.5%)
SAIO	13 (12.6%)	3 (23.1%)
Enterocutaneous fistula or pancreatic fistula-related problems	10 (9.7%)	1 (10%)
External stent, T-tube, or PTBD-related problems	9 (8.7%)	7 (77.7%)
Incisional hernia	8 (7.8%)	8 (100%)
POPH	7 (6.8%)	3 (42.8%)
Wound-related	9 (8.7%)	3 (33.3%)
DGE	6 (5.8%)	3 (50%)
HJ stricture-related	6 (5.8%)	4 (66.6%)
Gastritis-related	4 (3.9%)	3 (75%)
Stoma-related	2 (1.9%)	2 (100%)
Others	6 (5.8%)	2 (33.3%)

Readmissions within 30 days

Out of 103 patients, 53 (51.5%) were readmitted within 30 days after discharge. Intra-abdominal collection (n = 16, 30.2%) was the most common cause of readmission in this time frame. Of these 53 patients, 22 (41.5%) required intervention, with percutaneous drainage (PCD) and upper gastrointestinal endoscopy (UGIE) being the common procedures. Table 2 depicts the causes, interventions, and outcomes in patients who were readmitted within 30 days after discharge. The average hospital stay was 10.76 days (range = 1 to 48 days); 34 (64.1%) of 53 patients had good outcomes, whereas 19 (35.8%) of 53 had fair outcomes.

**Table 2:** Causes, interventions, and outcomes in patients readmitted within 30 days after discharge. SAIO = subacute intestinal obstruction; POPH = postoperative pancreatic hemorrhage; DGE = delayed gastric emptying; PTBD = percutaneous transhepatic biliary drainage; PCD = percutaneous drainage; UGIE = upper gastrointestinal endoscopy; DSA = digital subtraction angiography

Readmissions within 30 days		
Causes (total = 53)	Frequency	Percentage
Intra-abdominal collection	16	30.2%
SAIO	7	13.2%
Wound-related complications	6	11.3%
POPH	6	11.3%
DGE	6	11.3%
Enterocutaneous fistula or pancreatic fistula-related	3	5.7%
External stent-, T-tube-, or PTBD-related problems	3	5.7%
Gastritis-related problems	2	3.8%
Others	4	7.5%
Interventions (total = 22)	Frequency	Percentage
PCD	7	31.8%
UGIE	7	31.8%
Wound care	3	13.6%
Fluoroscopically guided tube repositioning	3	13.6%
DSA	2	9.1%
Outcomes (total = 53)	Frequency	Percentage
Good	34	64.1%
Fair	19	35.8%

Of these 53 patients, 27 (50.9%) were readmitted within seven days after discharge. The most common cause for readmission was intra-abdominal collection (n = 7, 25.9%) in these patients. Out of 27 patients, 12 (44.4%) required intervention, with PCD (n = 6, 50%) being the most common intervention. The average hospital stay of these patients was 12.2 days (range = 1-40 days); 24 of 27 (88.8%) patients had good outcomes, whereas three of 27 (11.1%) had fair outcomes.

Readmissions between 31 and 90 days

Out of 103 patients, 12 (11.6%) were readmitted between 31 and 90 days following discharge. External stent, T-tube, or percutaneous transhepatic biliary drainage (PTBD)-related problems (n = 4, 33.3%) were the most common causes of readmission in this time frame. Overall, four (30%) of 12 patients required intervention. Six (50%) of 12 patients had good outcomes. The average hospital stay was 19 days (range = 3-150 days). A detailed list of causes, interventions, and outcomes of this group is depicted in Table 3.

**Table 3:** Causes, interventions, and outcomes in patients readmitted between 31 and 90 days after discharge. PTBD = percutaneous transhepatic biliary drainage; SAIO = subacute intestinal obstruction; POPH = postoperative pancreatic hemorrhage; PCD = percutaneous drainage

Readmissions between 31 and 90 days		
Causes (total = 12)	Frequency	Percentage
External stent, T-tube, or PTBD-related problems	4	33.3%
Wound-related complications	2	16.7%
SAIO	2	16.7%
Intra-abdominal collection	2	16.7%
POPH	1	8.3%
Enterocutaneous fistula or pancreatic fistula-related	1	8.3%
Interventions (total = 4)	Frequency	Percentage
PCD	2	50%
Fluoroscopically guided tube repositioning	2	50%
Outcomes (total =12)	Frequency	Percentage
Good	6	50%
Fair	4	33.3%
Bad	2	16.7%

Readmissions after 90 days

Of 103 patients, 38 (36.9%) were readmitted after 90 days following discharge. Incisional hernia (n = 8, 21.1%) was the most common cause of readmission in this group, and 26 (68.4%) out of 38 patients underwent intervention during readmission. Other causes, interventions, and outcomes of this group are depicted in Table 4. These patients had an average hospital stay of 14 days (range = 1-120 days).

**Table 4:** Causes, interventions, and outcomes of patients readmitted after 90 days after discharge. HJ = hepaticojejunostomy; SAIO = subacute intestinal obstruction; PTBD = percutaneous transhepatic biliary drainage; PCD =  percutaneous drainage; UGIE = upper gastrointestinal endoscopy

Readmissions after 90 days		
Causes (total = 38)	Frequency	Percentage
Incisional hernia	8	21.1%
Strictured HJ	6	15.8%
Enterocutaneous fistula or pancreatic fistula-related	6	15.8%
Intra-abdominal collection	5	13.2%
SAIO	4	10.5%
External stent, T-tube, or PTBD-related problems	2	5.3%
Gastritis-related	2	5.3%
Stoma-related	2	5.3%
Wound-related complications	1	2.6%
Others	2	5.3%
Intervention (total = 26)	Frequency	Percentage
Hernioplasty	8	30.7%
Fluoroscopically guided HJ stricture dilatation	4	15.4%
PCD	4	15.4%
Laparotomy for bowel obstruction	3	11.5%
Fluoroscopically guided tube repositioning	2	7.7%
Restoration of bowel continuity	2	7.7%
UGIE	2	7.7%
Others	1	3.8%
Outcome (total = 38)	Frequency	Percentage
Good	23	60.5%
Fair	13	34.2%
Bad	2	5.2%

Factors predicting readmission

A previous history of cholangitis (OR = 1.771, 95% CI = 1.171-2.677, p = 0.007), postoperative fever (OR = 1.628, 95% CI = 1.081-2.452, p = 0.02), wound infection (OR = 2.011, 95% CI = 1.332-3.035, p = 0.001), and wound dehiscence (OR = 2.136, 95% CI = 1.333-3.423, p = 0.002) predicted readmission on univariate analysis, as shown in Table 5. On multivariate analysis, a previous history of cholangitis (OR = 1.755, 95% CI = 1.158-2.659, p = 0.008) and wound infection (OR = 1.995, 95% CI = 1.320-2.690, p = 0.001) independently predicted readmission, as shown in Table 6. Kaplan-Meier curves were used to analyze the impact of readmission on survival, and the difference between the two groups (p = 0.094) was not statistically significant.

**Table 5:** Univariate analysis of different variables predicting readmission after pancreaticoduodenectomy. POPF = postoperative pancreatic fistula; DGE = delayed gastric emptying

Variable	Odds ratio	95% confidence interval	P-value
Preoperative variables			
Age <60	1.051	0.665–1.662	0.830
Male gender	0.89	0.576–1.392	0.623
Preoperative jaundice of more than 10 mg/dL	1.721	0.733–4.042	0.213
Preoperative history of cholangitis	1.771	1.171–2.677	0.007
Medical risk factors affecting mortality	1.40	0.910–2.153	0.126
Preoperative biliary drainage	1.567	0.995–2.470	0.053
Intraoperative variables			
Type of pancreaticoduodenectomy (classical or pylorus-preserving )	0.902	0.599–1.358	0.620
Vascular resection	1.680	0.565–4.992	0.351
External stent for drainage of pancreaticojejunostomy	0.673	0.077–5.854	0.720
Internal pancreaticojejunostomy stent	1.200	0.795–1.812	0.385
Duration of surgery (six hours or less)	0.750	0.428–1.311	0.312
Soft consistency of the pancreas	0.867	0.576–1.305	0.493
Intraoperative blood transfusion	1.237	0.811–1.887	0.322
Benign histopathology	1.669	0.907–3.072	0.100
Postoperative variables			
Postoperative fever	1.628	1.081–2.452	0.020
Wound infection	2.011	1.332–3.035	0.001
Wound dehiscence	2.136	1.333–3.423	0.002
Postoperative bleeding	0.577	0.284–1.174	0.129
POPF	1.339	0.871–2.059	0.183
Intra-abdominal collection	1.351	0.855–2.134	0.197
External biliary fistula	1.396	0.731–2.663	0.312
DGE	0.817	0.498–1.341	0.424
Re-exploration in the postoperative period	0.987	0.534–1.824	0.967
Postoperative ascites	0.575	0.245–1.350	0.204
Postoperative hospital stay ≤10 days	0.434	0.488–1.361	0.434

**Table 6:** Multivariate analysis of different variables predicting readmission after pancreaticoduodenectomy.

Variable	Odds ratio	95% confidence interval	P-value
Preoperative history of cholangitis	1.755	1.158–2.659	0.008
Wound infection	1.995	1.320–3.017	0.001
Wound dehiscence	1.546	0.888–2.690	0.124
Postoperative fever	1.243	0.782–1.977	0.358

Our experience

**Figure 1:**
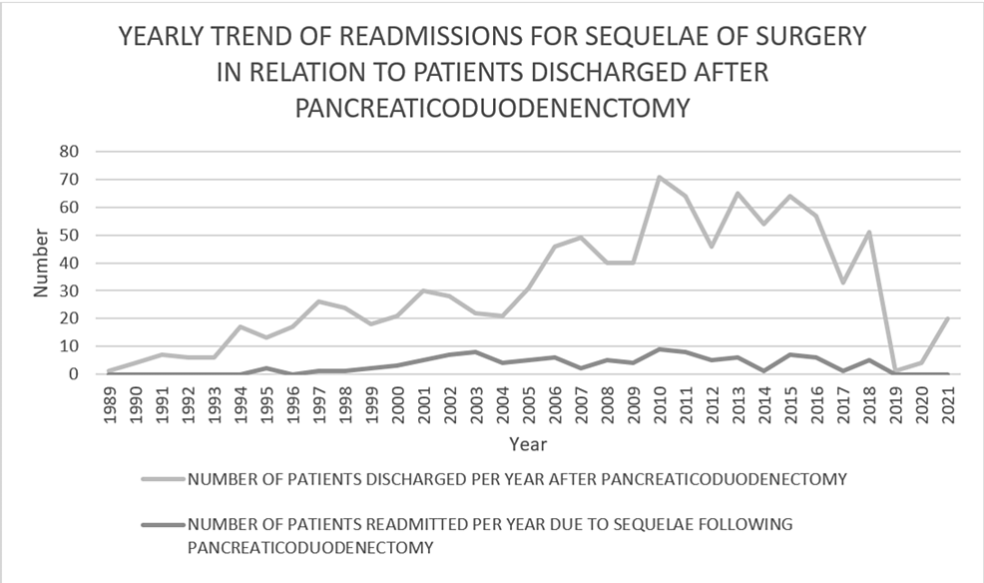
Plot showing the yearly trend of readmissions for the sequelae of surgery regarding patients discharged after pancreaticoduodenectomy (the drop in cases in 2019 was due to the COVID-19 pandemic).

The quinquennial readmission rate is depicted in Table 7. As the volume of patients discharged from our institute increased, our readmission rate for sequelae of surgery initially increased (the maximum five-year readmission rate was 11.8%) and then decreased. The yearly readmission rate is plotted in Figure 1.

**Table 7:** Readmission rate of patients following pancreaticoduodenectomy for sequelae of surgery: our institute’s experience represented quinquennially.

Time frame	Patients discharged after pancreaticoduodenectomy	Patients readmitted for sequelae of surgery (%)
1989 to 1993	24	0
1994 to 1998	97	4 (4.1%)
1999 to 2003	119	25 (21%)
2004 to 2008	187	22 (11.8%)
2009 to 2013	286	32 (11.2%)
2014 to 2018	259	20 (7.7%)
2019 to 2021 (the drop in numbers was due to the COVID-19 pandemic)	25	0

## Discussion

In our study, 10.3% of patients required readmission following PD, for causes related to sequelae of the index surgery, with intra-abdominal collection being the most common cause. Most of these readmitted patients (50.4%) required intervention. Overall, 61.2% of readmitted patients had good outcomes. The intra-abdominal collection was the most common cause of readmission within 30 days after discharge post-PD, with PCD and UGIE being the common interventions required. External stent, T-tube, or PTBD-related problems were the most common causes of readmission within 31 to 90 days following discharge. Readmission after 90 days following discharge was mostly due to an incisional hernia. In our study, factors such as a previous history of cholangitis and wound infection independently predicted readmission.

Readmissions after PD are attributed to multiple causes related to a primary disease, post-surgical complications, and adjuvant therapy. These readmissions can occur in different time frames. The readmission rate after PD varied from 11.6% to 59% [6-9]. This wide variation can be attributed to varied inclusion criteria in different studies. Our study focused on readmissions due to surgical sequelae following PD, with an overall readmission rate of 10.3% (103 out of 997) comparable to the reported range of 11.6% to 17% [9-11].

More than half of our readmissions (53 out of 103, 51.5%) occurred within 30 days of discharge. The 30-day readmission rate post-PD was highlighted by various studies [3,5-7,9,11-14]. Most of our patients in this group suffered sepsis-related complications (22 out of 53, 41.5%), comprising intra-abdominal collection (16 out of 53, 30.2%) and wound-related complications (6 out of 53, 11.3%), similar to other studies [3,6,10,12,14]. Some studies [5,7] reported nutritional or gastrointestinal problems as the most common causes of 30-day readmission. In our study, high intervention rates (22 out of 53, 41.5%) and good outcomes (34 out of 53, 64.1%) with no mortality in patients readmitted within 30 days following discharge signified the need for frequent follow-ups in the first month after discharge.

About half (27 out of 53, 50.9%) of the patients who required readmission within 30 days were readmitted within seven days after discharge. This group had a high intervention rate (12 out of 27, 44.4%) and most patients had good outcomes (24 out of 27, 88.8%). Fong et al. [15], also determined that 50% of all readmissions occurred within seven days after discharge (early readmission) with ileus, DGE, and pneumonia as the most common causes. Ahmad et al. [12] reported that 6% of their readmissions occurred within seven days and believed that “these early readmissions (readmission within seven days) could be avoided with proper anticipation of complications and by attaching skilled nursing staff to these patients.” We followed our patients with postoperative complications within seven days after discharge, which helped us identify patients who may have needed life-saving intervention. Early follow-ups can avoid mortality, as reflected in our study. In resource-poor centers such as ours, educating patients’ caretakers during hospital stays almost compensated for the lack of skilled nursing staff.

Of those readmissions (12 out of 103, 11.6%) that occurred between 31 and 90 days following discharge, 50% (six of 12) were related to external stents, T-tubes, or PTBD-related problems and wound-related complications, as depicted in Table 3. Judicious use of stents and PTBD can prevent readmissions related to complications such as blockage, slippage, and dislodgement. Failure to thrive was found to be a common cause of readmissions between 31 and 90 days in some studies [12,13]. Nutritional education for patients and their caretakers can help address this problem.

Very few studies focused on the long-term causes of readmissions following PD. Of our readmissions, 36.9% (38 out of 103) occurred 90 days following discharge, and the most common cause was incisional hernia (eight out of 38, 21.1%), as found in other studies [10]. Most of the patients (60.5%) readmitted after 90 days after discharge had good outcomes in our study, as depicted in Table 4. Kastenberg et al. [5] noted readmission rates of 27.4% within six months and 37.4% within one year of discharge, but the causes of readmission were not mentioned. Reddy et al. [6] reported that 25% of their late readmissions (30 days to within 1 year) were related to operative complications. Hari et al. [16] studied 90-day readmission rates after PD and found dehydration and malnutrition to be the major causes of readmission. This study determined that “self-care education” provided to patients and attendees could decrease preventable readmissions and that the best time for this self-care education would be in the preoperative period.

Kent et al. [17] found that 14% of their patients were readmitted for some clinical concern but did not find any complications on workup. Kastenberg et al. [5] reported 8% of patients with negative diagnostic workups. We have an “observation room facility” at our department, where patients are monitored and reassessed after six to eight hours with biochemical and imaging workups, which helps avoid unnecessary readmissions.

The intervention requirement and outcomes during readmissions have not been studied previously. Our study showed an overall intervention rate of 50.4% (52 out of 103), and 61.2% (63 out of 103) patients had good outcomes. Only 3.9% (four out of 103) had bad outcomes. Readmitted patients had high intervention requirements, some even life-saving. If treated effectively, the results should be satisfactory. Readmissions should not be considered as a burden on the healthcare system because they provide an opportunity to improve patient care.

Patient-related factors such as age, gender, and comorbidities were not associated with readmission risk in our study, consistent with other studies [5,12,15,17-19]. Some studies [9,14,20] reported higher readmission rates in younger patients, which may be attributed to “extensive dissections” performed in younger patients leading to increased complications. Although some studies [11,14,21,22] reported readmissions to be significantly associated with patients with comorbidities, we found no such association in our study. These variations in different studies could be due to differences in samples.

Multiple preoperative factors contribute to readmission rates following PD. Patients who experienced cholangitis in the preoperative period were associated with an increased risk of readmission in our study (OR = 1.755, 95% CI = 1.158-2.659, p = 0.008). Although not found in our study, it has been reported that patients with chronic pancreatitis had an increased association with readmission following PD due to recurrent pain episodes [12,18]. Preoperative biliary drainage was not associated with readmission in our study as supported by others [12,18,20]. Intraoperative factors such as small pancreatic ducts [13,17] and vascular resections [15,20] were identified to be risk factors for readmission. Such patients are prone to increased postoperative complications and need a properly planned discharge with early follow-up. Postoperative complications were reported to be a predictor of readmission in many studies [5,7,9,12,14,17,20], but the complications enumerated in these studies were diverse. Postoperative fever (OR = 1.628, 95% CI = 1.081-2.452, p = 0.02), wound infection (OR = 2.011, 95% CI = 1.332-3.035, p = 0.001), and wound dehiscence (OR = 2.136, 95% CI = 1.333-3.423, p = 0.002) predicted readmission in our study on univariate analysis, as shown in other studies [7,12]. POPF [9,12,15,17], DGE [17], and intra-abdominal abscess [7,12] were also important predictors of readmission.

The patients’ condition at the time of discharge was studied to predict readmission in some studies. “Non-routine discharge (discharge with home health services or discharge to nursing or rehabilitation facility),” as described by Kastenberg et al. [5], was associated with 30-day readmission (OR = 2.69, 95% CI = 1.17-6.12). The presence of pancreatic leak [23] at discharge and discharge with drain [13] were also predictors of readmission.

Does the length of the hospital stay during the index surgery predict readmission? In our study, patients who required readmission had significantly longer postoperative hospital stays during their index surgery (mean = 20.74 days, SD = 14.35) compared to those not readmitted (mean = 17.74 days, SD = 11.61, p = 0.014). Prolonged hospital stays during index surgery have been identified as a risk factor for readmission by many studies [3,6,7,11,13,14,20]. Patients who require a prolonged hospital stay during an index procedure are the ones who suffer complications, and it is imperative to be aware that these patients might return after discharge.

In our study, early discharge (discharge within 10 days after surgery) was not found to affect readmission (OR = 0.434, 95% CI = 0.488-1.361, p = 0.434), as reported in other studies [7,17,24]. Balzano et al. [24] found that patients with a “fast track perioperative care program” after PD had shorter hospital stays (p < 0.001) but no significant change in the readmission rate (7.1% versus 6.3%, p = 0.865). Hospitals should develop their own criteria for discharge, and patients fulfilling those criteria can be discharged as early as possible, without any fear of readmission. Ceppa et al. [25] showed that a “reengineered discharge checklist” decreased the readmission rate from 23% to 15-19%. We discharge patients when they are sepsis-free, have well-established nutritional access, and are compliant with discharge advice. Patients’ caretakers have an important role to play, so they should be well-educated about nutrition, wound care, and warning signs in the early postoperative period. Many studies focused on the effect of readmission on the survival of patients. Although some studies [26] showed decreased survival in patients who required readmissions, in our study, readmissions did not affect survival, similar to other studies [5,12,17].

Many studies [3,4,21] have focused on the effect of hospital volume on outcomes for patients undergoing PD. Some centers [2] showed improved patient care and outcomes as their experience in performing PD increased. Patients requiring PD should be referred to high-volume centers to lower readmissions and improve healthcare [3]. High-volume institutions should formulate steps for tackling readmissions in this era of modern surgery. In our institution, as the volume of PD increased, the readmission rate increased initially (the highest five-year readmission rate was 21%). With improved postoperative care and well-planned discharge policies, the readmission rate decreased (7.7%) despite an increased volume (Table 7, Figure 1). We identify and enroll one caretaker (from patients’ attendants) for each patient who undergoes PD and train them in wound care, nutritional support, and warning symptoms from the early postoperative period. This step has prevented nutrition-related readmissions. Well-educated caretakers bring patients with warning symptoms early to the hospital, decreasing mortality.

Our study focused on patients who were readmitted for both early and late consequences following PD, whereas most previous studies focused only on early consequences. We were unique in reporting the intervention rate of readmissions and outcomes of patients readmitted in different time frames, which most previous studies have not focused on. We included a homogenous sample of patients who were readmitted for sequelae after PD.

The study had its own demerit in that it was retrospective. The study period was of more than three decades (1989 to 2021). Over this long duration, the changes in patient approach, surgical techniques, postoperative care, and hospital policies might influenced our results. The underreporting of readmission rates is a concern in hospital-based studies [6] because some readmissions can occur in secondary hospitals [11]. As a tertiary referral center, our surgical patients who required readmission were referred back to our institution, but readmissions in secondary hospitals in our study are a possibility. Because patients in emergencies might not have had access to our hospital, a major limitation of this study is that our readmissions might have been underestimated.

## Conclusions

Readmission for sequelae after PD is a significant health problem. Most of these readmissions occur within 30 days after discharge. Most of these patients require intervention, and some interventions can be life-saving. Readmitted patients have good outcomes despite the need for intervention. Patients who suffered postoperative complications are at a high risk of readmission and frequent follow-ups in these patients can decrease them. A well-planned discharge and properly designed post-discharge care systems can help reduce readmissions. Patients’ caretakers should be educated about nutrition management and wound care, and this education should be started in the early postoperative period. Readmissions should not be considered a scale for poor healthcare because they help decrease 30-day post-discharge mortality.
